# The Homœopathic Therapeutics of Diarrhœa, Dysentery, Etc.

**Published:** 1897-06

**Authors:** 


### BOOK NOTICES.

THE HOMEOPATHIC THERAPEUTICS OF DIARRHEA, DYSENTERY, CHOLERA, CHOLERA MORBUS, CHOLERA INFANTUM, AND ALL OTHER LOOSE EVACUATIONS OF THE BOWELS. By James B. Bell, M. D. Fourth edition. Philadelphia: Boericke & Tafel, 1011 Arch Street. 1897. Price, cloth, $1.50 net. By mail, $1.58.

This justly celebrated monograph was first published in 1869. It is incomparably the best monograph ever published by a disciple of our school. At the time of its first issue it was a revelation in the making of repertories for its clearness of arrangement, not alone in classification of symptoms, but in the use of type to make those symptoms available quickly and surely. When it first came out the writer of this review was a beginner in practice. He was treating a sufferer from chronic diarrhea contracted in the gold fields of California, and therefore of nearly twenty years, continuance. The patient had offered the writer a prize of a considerable sum of money over his regular fee if he could cure this diarrhea. While engaged in the task Dr. Bell's book appeared and we immediately secured a copy of it, studied it carefully while treating the case, and ultimately found, through its aid, the simillimum, triumphantly cured the case, and won the prize. Since then we have always felt a particular regard for the book and it has frequently helped us in the treatment of obscure and obstinate cases of diarrhea during the past twenty-eight years. In all our journeys for recreation we have invariably carried a copy of the book in our luggage. We have always warmly recommended the book to all students and beginners of medicine, and have even presented copies of it to friends in the profession.

Some disappointment was felt when the third edition came out because of the largely increased size of the pages, which made it rather unwieldy to carry. This has been corrected in this fourth edition and the size of page reduced to the former small octavo, and that, too, without sacrificing the legibility of the type or cutting off any of the valuable material of which the book is made.

Dr. Bell says in his fourth preface: "The most that can be said as a preface to a fourth edition, is that a thorough revision and re-revision, and a renewed comparison with all the materia medica now available, reveals but few changes to make, and no remedies to add or to omit."

We cannot forbear adding the rest of the preface:

"*Allen's Symptom Register* gives four hundred and twenty-five remedies as having diarrhea, and *Knerr's Repertory of the Guiding Symptoms* a much smaller list, but none of them not already included in this book are suited for a place in it, either because the proving is indefinite, or because the diarrhea is simply accessory to a larger and more important group of symptoms (as in Diadema in intermittent fever, or Asterias-rubens in epilepsy, or Arum-triyphyllum in typhoid or scarlet fever), and is not particularly well defined in itself. It would seem, therefore, that this little work is now as complete as it can well be made, for at least some time to come.

"Homeopathy is not making that kind of 'progress' that renders a whole medical library obsolete every ten years, but instead of that, is all the time laying up in its storehouse treasures new and old."

As a closing word we once more, as we have ever in print and in speech, cordially recommend this excellent monograph to all true homeopathic physicians.

